# Editorial: Women in science: strengthening rehabilitation in health systems

**DOI:** 10.3389/fresc.2023.1221798

**Published:** 2023-07-04

**Authors:** Merce Avellanet, Christina-Anastasia Rapidi

**Affiliations:** ^1^ Research Group in Health Sciences and Health Services, Universitat D'Andorra, Sant Julià de Lòria, Andorra; ^2^ Rehabilitation Department, Hospital Nostra Sra de Meritxell, Escaldes-Engordany, Andorra; ^3^ PRM Department, General Hospital “G.Gennimatas”, Athens, Greece

**Keywords:** women, science, research, rehabilitation, health system

**Editorial on the Research Topic** Women in science: strengthening rehabilitation in health systems

Frontiers in Rehabilitation Sciences underline their concern on gender gap in science and offered a platform to promote the work of women scientists. While less than 30% of researchers worldwide are women according to Unesco Institute of Statistics, 82% of the authors who published in this topic are women (27 authors: 22 women and 5 men).

Health conditions leading to severe levels of disability present increased prevalence due to the increased population of aged people, increased number of persons with disabling musculoskeletal diseases and of survivors with neurological, cardiovascular, and neoplasm conditions (1). According to the latest *Global Report on Health Equity for Persons with Disabilities* (2022) of the WHO “approximately 1.3 billion people (16% of the population) have significant disability” (2). However, many more people could benefit from rehabilitation services, approximately 2.41 billion people (at least one in every three people in the world) need rehabilitation at some point during a disease or injury (3). Despite this data, health-related rehabilitation services remain under-funded, under-researched, and under-provided (3).

A special collection of 5 articles deal with different perspectives of rehabilitation services, disability, and health care. Specifically, the articles refer to a Physical and Rehabilitation Medicine (PRM) clinical point of view, to a team approach and to qualitative research.

From a PRM clinical point of view, a clinical approach focused on the importance of PMR diagnosis: Ultrasound evaluation, by Yuan et al.

From a team approach, the case report of an elderly woman with tetraplegia and sarcopenia due to spinal tuberculosis focused on the importance of functional outcomes and QoL, by Defi et al.

From qualitative research in other articles (Lampart et al. Hale et al. Feldner et al.): the goal setting process in acute SCI emphasized the importance of strong collaboration between different professionals, communication abilities to educate patients with acute traumatic or non-traumatic SCI as well as planning therapies within a true patient-centered perspective by Lampart et al.

Qualitative sociological research tries to engage participation of persons with type 2 diabetes. This research addresses aspects of a Community Exercise Programme that attendees and healthcare providers considered to be important because of their person-centered approach by Hale et al.

These two papers based on qualitative research, highlight as well the fundamentals of team approach.

Finally, Feldner et al. envision societal views toward disability and through qualitative research, they focus on social inclusion. During the pandemic period, disabled people were faced to critical barriers that they continue to address. Consequently “allyship” was considered the word of the year for 2021 and “Ableism”: discrimination in favor of able-bodied people. This qualitative, phenomenological study includes students, faculty, and staff on the University of Washington campus who identify themselves as disabled/with a disability. Participants share their experiences of ableism and allyship in different roles such as patients, providers, trainees, or educators, revealing the ubiquity of ableism in healthcare. The authors stated that “the study is one of the first of its kind in the US to use a CDS framework to understand lived experiences of ableism and allyship across faculty, staff, and students in a university setting, and to subsequently apply these experiences to inform the development of more inclusive practices in rehabilitation education in a post-COVID era” (Feldner et al.).

Ιt is impressive that significant supply and demand gaps were found even in high-income and not just lower-income countries where rehabilitation services are presumed to be lacking. These discrepancies were enlarged during COVID-19 pandemic. There is an urgent need to promote and further develop the rehabilitation services in every level of health care from the primary care level to the tertiary care levels (1, 3).
